# Viability of *Metarhizium anisopliae *conidia (metsch.) *Sorok *preserved in packages containing silica gel

**DOI:** 10.1186/1753-6561-8-S4-P128

**Published:** 2014-10-01

**Authors:** Aline de Freitas, Maria Gabriela Bispo Almeida Araújo, Eleci Adriano Hendges, Ana Gorete Campos de Azevedo, Danielle Marques de Oliveira Lima, Iedo Silva da Cruz, Marcelo da Costa Mendonça, Leandro Eugenio Cardamone Diniz

**Affiliations:** 1 Universidade Tiradentes, Aracaju, SE, Brazil; 2 Emdagro/FAPITEC, Aracaju, SE, Brazil; 3 Embrapa Tabuleiros Costeiros, Aracaju, SE, Brazil

## Background

The entomopathogenic fungi are important microbial agents for their efficient performance as microbial biopesticides in pest control. The development of commercial products formulated with entomopathogenic fungi is in a promising market for biopesticides. However, the fungi can lose their viability when exposed to unfavorable conditions of temperature, humidity and ultraviolet radiation, affecting the life of conidia. Among these constraints, there is the relative humidity through the presence of free water in formulated as one of the main factors that affect the viability of the organism in the form and function of storage time. The use of desiccant agents, such as silica gel, may be a viable alternative to favor the retention of moisture in the air, reducing the adsorption of water molecules by the system. Thus, the aim of this study was to determine the efficiency of silica gel in maintaining the viability of dry conidia of *Metarhizium anisopliae*.

## Methods

The treatments consisted of 0.02 g of dry conidia of *M. anisopliae *with initial viability of 95.5%. The fungi were stored in microcentrifuge tubes with three replicates, containing different amounts of silica gel as drying agent (0.15 g, 0.30 g and 1 g) and a control containing only the conidia. The treatments were stored in chambers of BOD controlled temperature of 26º ± 1.0 °C. The viability of conidia was assessed in plates Rodac once a month for 180 days, according to the methodology described by [1].

## Results

It was observed that the treatments containing silica, independent of the amount (0.15 g, 0.30 g and 1g) with high spore viability when compared to the control without the presence of silica gel. Since the increase of the silica also maintained a higher viability, since silica is a product which retains moisture in the air by physical adsorption, or a process in which water molecules are trapped in the surface pore desiccants.

## Conclusion

The presence of silica gel throughout the analysis period considerably aided in maintaining the viability compared to treatment without the silica gel since it decreases the moisture present in the system increasing the shelf.
